# Switching to lamivudine plus darunavir/r dual therapy in a cohort of treatment-experienced HIV-positive patients: the experience of an Italian centre

**DOI:** 10.7448/IAS.17.4.19817

**Published:** 2014-11-02

**Authors:** Alberto Borghetti, Annalisa Mondi, Benedetta Piccoli, Roberta Gagliardini, Silvia Lamonica, Nicoletta Ciccarelli, Alessandro D'Avino, Federico Pallavicini, Roberto Cauda, Andrea De Luca, Massimiliano Fabbiani, Simona Di Giambenedetto

**Affiliations:** Institute of Clinical Infectious Disease, Catholic University of Sacred Heart, Rome, Italy

## Abstract

**Introduction:**

According to recent evidence about boosted protease inhibitors (PIs/r)-simplified regimens, the combination of 3TC and DRV/r 800/100 mg could represent a feasible option for optimizing antiretroviral therapy (ART) in treatment-experienced HIV+ patients.

**Patients and Methods:**

We retrospectively evaluated patients switching to 3TC+DRV/r, with at least six months of viral suppression, no resistance mutation to DRV or 3TC and not HBV-coinfected: incidence of ART discontinuation and of virological failure (VF: 2 consecutive HIV-RNA determinations>49 cps/mL or a single one≥1000 cps/mL) and the probability of remaining discontinuation-free during one-year follow-up (FU), as well as changes in laboratory parameters at 1, 3, 6 and 12 months were estimated.

**Results:**

We included 94 patients: 74 males, mostly MSM (39.4%), with 49 years old, 9 years of HIV disease, 8 years of ART (median values). Median nadir CD4 count and zenith viral load (log10) were 194 cells/µL and 4.90, respectively. Ten patients were HCV-coinfected and 38 had at least a previous VF. Seventy-four patients were on an NRTIs-based triple regimen (mainly TDF/FTC or 3TC/ABC) whereas 14 on another PI-based dual therapy (mainly LPV/r). Incidence of treatment discontinuation was 12.4 per 100 patients-year follow-up (PYFU), but only 2 patients experienced a VF (3.5 per 100 PYFU). Mean time free from discontinuation was 5 years (95% CI 4–6), with a cumulative one-year estimated probability of staying on 3TC+DRV/r of 85.9%. At three months, a trend of increased CD4 cells count (+42 cells/µL, p 0.059) was observed, but not confirmed at later time point; an increase of total cholesterol (TC, +17mg/dL, p 0.008) and LDL (+19 mg/dL, p 0.002), and a decreased level of AST and ALT (−2 UI/L, p 0.045; −5 UI/L, p 0.009, respectively) were also detected. Total bilirubin was reduced (−0.71 mg/dL, p 0.038). At 6 and 12 months, alteration of lipid profile was similar, with also an increased TC/HDL ratio (+0.48, p=0.030, at six months) and HDL/LDL ratio (−0.04, p=0.035, at 12 months). A significant decrease in ALT levels (−6 UI/L, 0.013) and a diminishing trend for AST and total bilirubin, as well as a significant increase in renal function (GFR +4mL/min, p 0.048) were observed at 12 months.

**Conclusions:**

These observations on 3TC+DRV/r-based dual therapy simplification in virologically suppressed patients show a good profile of efficacy and safety. An extended FU time is needed in order to establish the real impact of this promising therapeutic choice.

